# Coronary stent infection causing perforation and thrombotic occlusion after early post-PCI methicillin-resistant *Staphylococcus aureus* bacteraemia: an autopsy case report

**DOI:** 10.1093/ehjcr/ytag671

**Published:** 2026-09-08

**Authors:** Shota Hirai, Goro Fujiki, Atsushi Nakagawa, Taichi Okabe

**Affiliations:** Department of Cardiology, Nippon Life Hospital, 2-1-54, Enokojima, Nishi-ku, Osaka 550-0006, Japan; Department of Cardiology, Nippon Life Hospital, 2-1-54, Enokojima, Nishi-ku, Osaka 550-0006, Japan; Department of Cardiology, Nippon Life Hospital, 2-1-54, Enokojima, Nishi-ku, Osaka 550-0006, Japan; Department of Cardiology, Nippon Life Hospital, 2-1-54, Enokojima, Nishi-ku, Osaka 550-0006, Japan

**Keywords:** Coronary stent infection, *Staphylococcus aureus* bacteraemia, Drug-eluting stent, Methicillin-resistant *Staphylococcus aureus*, Case report

## Abstract

**Background:**

Coronary stent infection (CSI) is rare but can be fatal. In *Staphylococcus aureus* bacteraemia (SAB), treatment duration is guided by classification as uncomplicated or complicated SAB. However, whether a recently implanted drug-eluting stent (DES) should be regarded as intravascular prosthetic material remains unclear.

**Case Summary:**

An 83-year-old woman underwent percutaneous coronary intervention (PCI) for a severely calcified right coronary ostial lesion. After rotational atherectomy, a DES was implanted, and final angiography showed adequate expansion. On hospital day 7, cellulitis developed at a peripheral intravenous catheter site. Blood and skin cultures grew methicillin-resistant *Staphylococcus aureus* (MRSA). Intravenous linezolid was started because of renal dysfunction. The episode was considered to be uncomplicated SAB because follow-up blood cultures were negative, fever resolved, transthoracic echocardiography showed no vegetation, and no metastatic infection was identified. Antimicrobial therapy was stopped after 14 days. Fever and inflammatory markers did not recur, but nonspecific chest symptoms persisted. Follow-up coronary angiography showed no clear abnormalities. After discharge, MRSA bacteraemia recurred. The patient developed shock with inferior ST-segment elevation, followed by pulseless electrical activity and death. Autopsy revealed coronary wall disruption, surrounding haemorrhage, ostial thrombotic occlusion, gram-positive cocci, and neutrophilic infiltration near the right coronary ostial stent, leading to the diagnosis of CSI.

**Discussion:**

This case raises the possibility that a recently implanted DES may serve as an occult endovascular focus during SAB. Negative follow-up blood cultures, defervescence, normalized inflammatory markers, and unrevealing imaging may not exclude CSI; persistent or recurrent chest symptoms after recent PCI should prompt targeted reassessment.

Learning points

- A recently implanted drug-eluting stent may serve as an occult endovascular focus during *Staphylococcus aureus* bacteraemia.
- Negative follow-up blood cultures and unrevealing initial imaging may not exclude coronary stent infection.
- Persistent or recurrent chest symptoms after recent PCI in patients with *S. aureus* bacteraemia should prompt targeted reassessment for coronary stent infection.

## Introduction

Coronary stent infection (CSI) is exceedingly rare but can be fatal because of coronary aneurysm, perforation, or stent thrombosis.^1–3^ In *Staphylococcus aureus* bacteraemia (SAB), treatment duration is guided by infective endocarditis, metastatic infection, persistent bacteraemia, and non-removable prosthetic material.^4,5^ However, whether a recently implanted drug-eluting stent (DES) should be considered an intravascular prosthetic material in SAB risk assessment remains unclear. We report an autopsy case of methicillin-resistant *Staphylococcus aureus* (MRSA) bacteraemia that initially appeared consistent with uncomplicated SAB but was ultimately associated with DES infection, coronary perforation, and thrombotic occlusion of the coronary ostium.

## Summary figure

**Figure:**
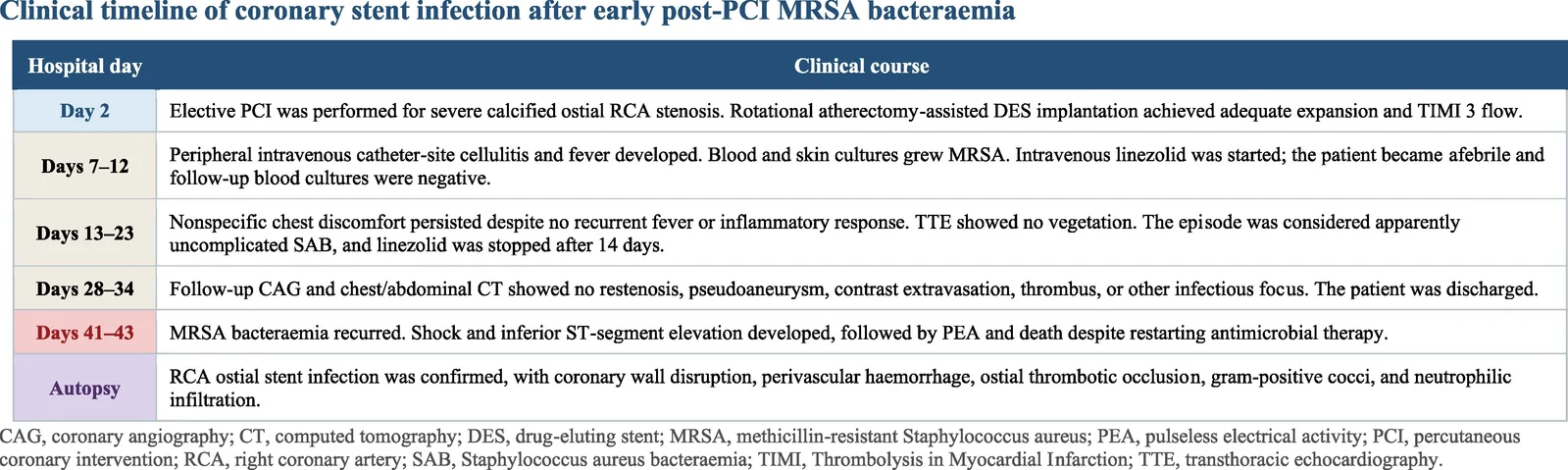
Clinical timeline of coronary stent infection after early post-PCI methicillin-resistant *Staphylococcus aureus* (MRSA) bacteraemia. The timeline summarizes elective PCI for a right coronary ostial lesion, subsequent MRSA bacteraemia associated with peripheral intravenous catheter-site cellulitis, persistent chest discomfort despite apparently reassuring follow-up investigations, recurrent MRSA bacteraemia with inferior ST-segment elevation and death, and autopsy-confirmed right coronary ostial stent infection with coronary wall disruption and thrombotic occlusion.

## Case presentation

An 83-year-old woman with hypertension, dyslipidaemia, type 2 diabetes, chronic kidney disease, and previous percutaneous coronary intervention (PCI) of the left anterior descending artery was admitted for elective PCI. She developed exertional chest pain, and coronary computed tomography (CT) angiography suggested severe proximal right coronary artery (RCA) stenosis. Coronary angiography (CAG) confirmed 90% ostial RCA stenosis without other significant coronary lesions, and elective PCI was planned. Laboratory testing showed no inflammatory response (white blood cell count, 5390/μL; C-reactive protein, 0.36 mg/dL), anaemia (haemoglobin, 9.4 g/dL), and impaired renal function (creatinine, 1.15 mg/dL; estimated glomerular filtration rate, 34.6 mL/min/1.73 m^2^).

**Figure 1:**
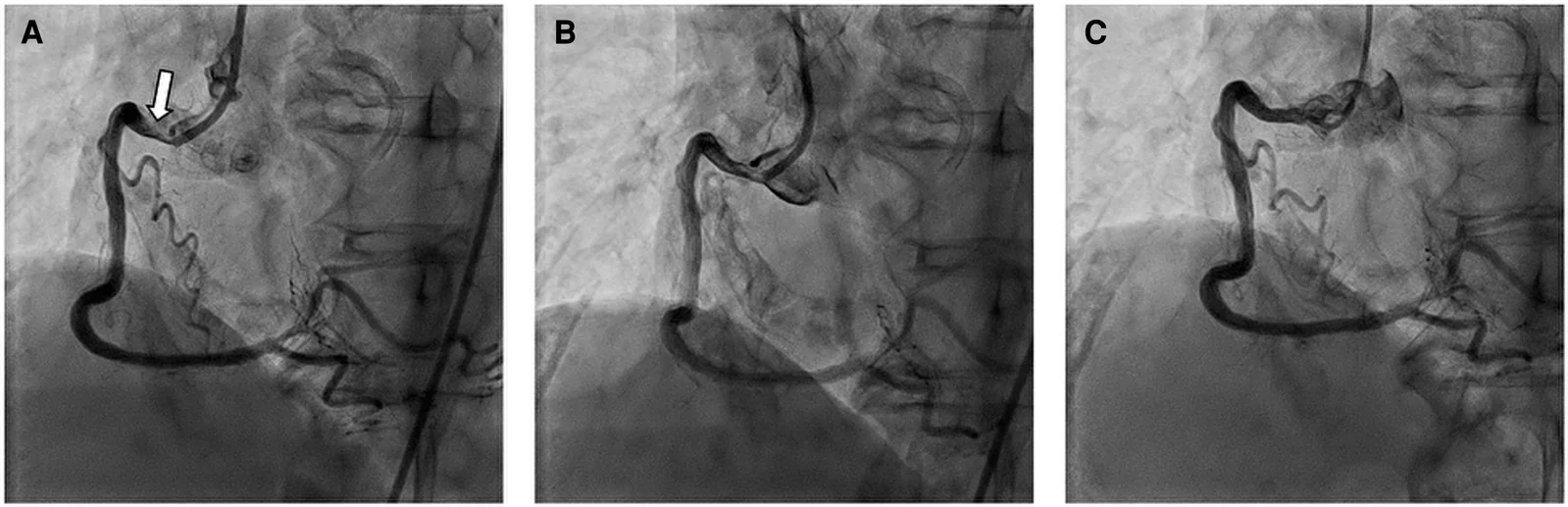
Coronary angiographic findings during index PCI and follow-up evaluation. (*A*) Baseline coronary angiography showed severe stenosis at the ostium of the right coronary artery (arrow). (*B*) After rotational atherectomy-assisted drug-eluting stent implantation, final angiography showed adequate lesion expansion with TIMI 3 flow and no apparent periprocedural complication. (*C*) Follow-up coronary angiography performed for persistent non-specific chest symptoms showed no restenosis, pseudoaneurysm, contrast extravasation, or visible thrombus at the stented segment.

On hospital day 2, PCI was performed through the right femoral artery using a 6F sheath, given previous difficulty with radial access and the anticipated need for rotational atherectomy. Intravascular ultrasonography (IVUS) showed severe stenosis with semicircumferential calcification and a focal calcified nodule. Rotational atherectomy with a 1.75-mm burr was followed by balloon predilation. After IVUS confirmed lesion expansion, a 3.5 × 18 mm everolimus-eluting stent was deployed with approximately 1 mm protruding into the aorta and post-dilated. Final angiography showed adequate expansion and thrombolysis in myocardial infarction (TIMI) 3 flow without complications (*Figure 1A, B*). Aspirin and clopidogrel had been started 2 weeks before PCI.

Discharge had been planned for day 4, but worsening anaemia (haemoglobin, 7.5 g/dL) and renal dysfunction (creatinine, 1.36 mg/dL) on day 3 prompted continued intravenous hydration and observation. On day 5, pain at the left forearm peripheral intravenous catheter site prompted catheter removal and replacement. On day 7, fever, erythema, and swelling developed at the previous site. Cefazolin was started after two sets of blood cultures were obtained. Although the patient became afebrile on day 9, blood and skin cultures grew MRSA on day 10. Because of impaired renal function and concern regarding vancomycin-associated nephrotoxicity, intravenous linezolid 600 mg every 12 h was started. The cellulitis improved without drainage. Two sets of follow-up blood cultures on day 12 were negative, and transthoracic echocardiography (TTE) on day 15 showed no vegetation. With no evidence of metastatic infection, the episode was considered uncomplicated SAB. Linezolid was discontinued on day 23 after 14 days of therapy.

**Figure 2:**
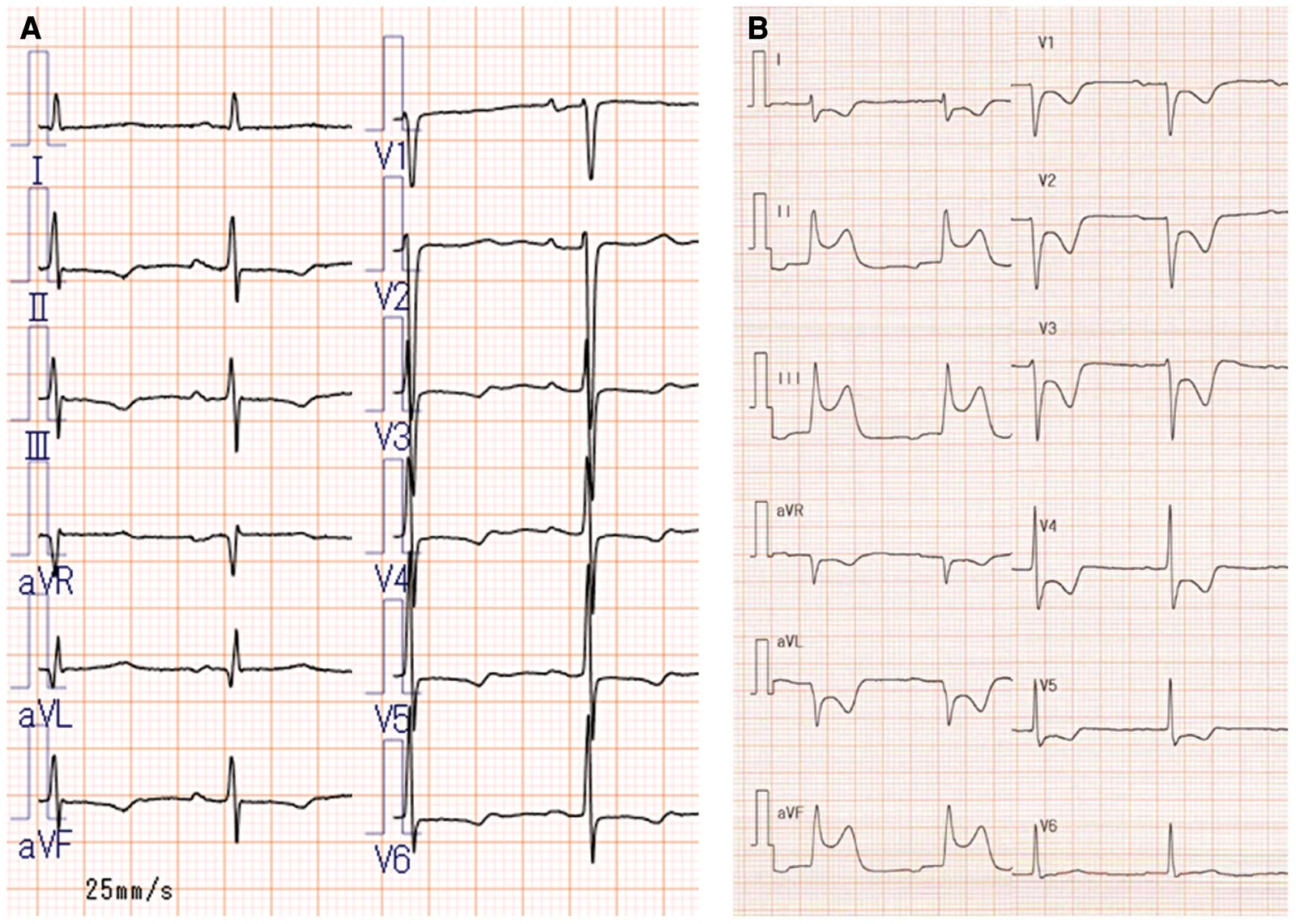
Baseline and terminal electrocardiograms. (*A*) Admission electrocardiogram. (*B*) During shock, new ST-segment elevation appeared in leads II, III, and aVF, consistent with acute inferior myocardial ischaemia.

From approximately day 13, intermittent chest discomfort persisted without recurrent fever or inflammatory response. CAG on day 28 showed no restenosis, pseudoaneurysm, contrast extravasation, or thrombus (*Figure 1C*; Supplementary material online, *Video S1*), and CT on day 29 showed no infectious focus. She remained afebrile without inflammatory marker elevation but with intermittent chest discomfort and was discharged on day 34. Readmitted on day 37 for dizziness, she remained afebrile without inflammatory marker elevation, while chest discomfort persisted. On day 40, her temperature increased to 37.5°C; on day 41, the white blood cell count and C-reactive protein increased to 11 000/µL and 6.0 mg/dL, respectively, and blood cultures again grew MRSA. Despite restarting antimicrobial therapy, shock and inferior ST-segment elevation developed (*Figure 2*), followed by pulseless electrical activity and death.

**Figure 3:**
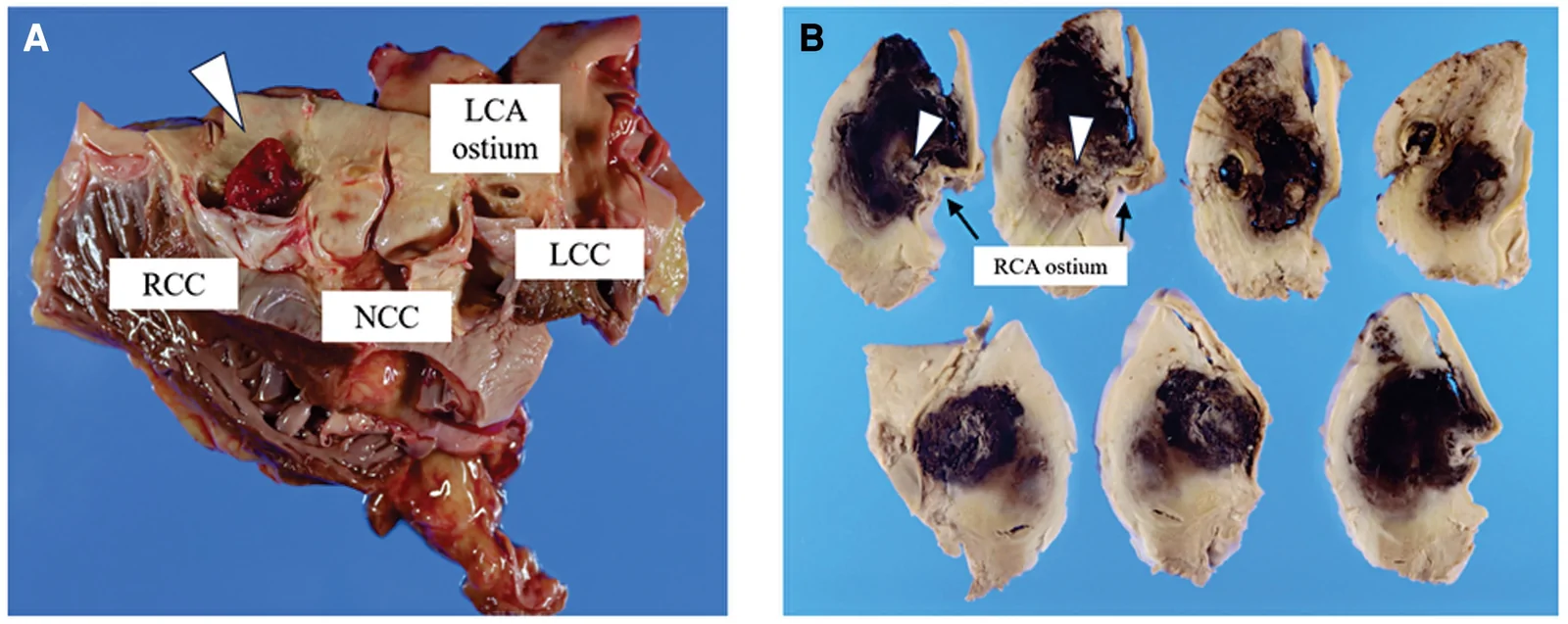
Gross autopsy findings of the right coronary ostial stent. (*A*) Gross examination showed thrombotic occlusion at the coronary ostium (white arrowhead). (*B*) Disruption of the coronary wall was observed near the stented ostial segment of the right coronary artery (white arrowheads). The black arrows indicate the RCA ostium. The surrounding dark haemorrhagic area in the epicardial adipose tissue was consistent with perivascular haemorrhage. LCA, left coronary artery; LCC, left coronary cusp; NCC, non-coronary cusp; RCA, right coronary artery; RCC, right coronary cusp.

**Figure 4:**
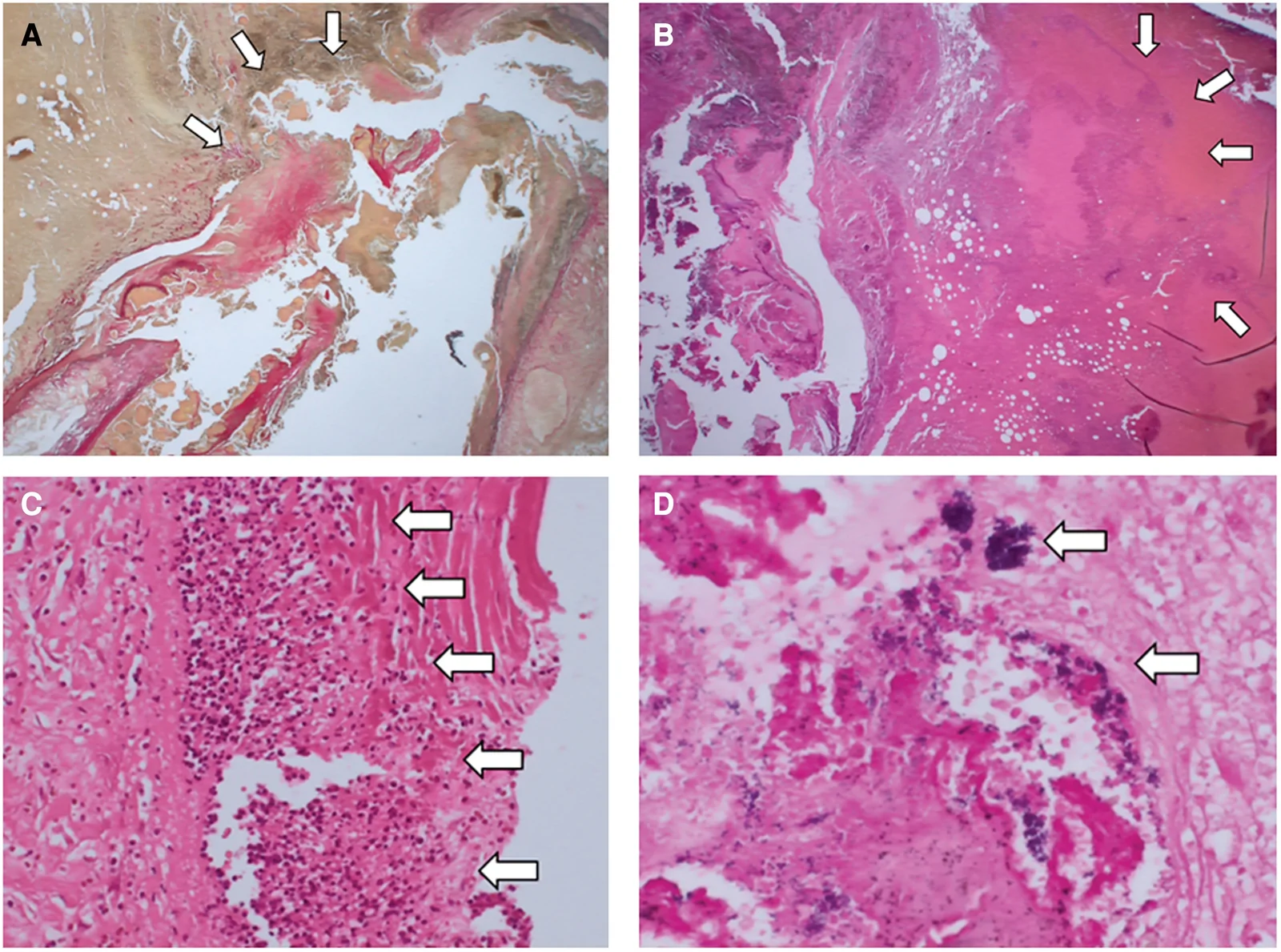
Histopathological findings confirming coronary stent infection. (*A*) Elastica van Gieson staining showed disruption of the coronary wall near the right coronary ostial stent (arrows). (*B*) H&E staining showed perivascular haemorrhage around the disrupted coronary wall (arrows). (*C*) High-power H&E staining showed dense neutrophilic infiltration extending into the surrounding tissue (arrows). (*D*) Gram staining demonstrated gram-positive cocci within the inflamed tissue (arrows). These pathological findings supported the diagnosis of coronary stent infection. H&E, Haematoxylin and eosin.

Autopsy showed coronary wall disruption, surrounding haemorrhage, and thrombotic ostial occlusion at the stented RCA segment (*Figures 3A, B* and *4A, B*). Histology revealed neutrophilic infiltration extending from the coronary wall into the surrounding tissue, with gram-positive cocci (*Figure 4C, D*). These findings supported coronary stent infection causing coronary perforation and thrombotic ostial occlusion.

## Discussion

The key lesson is that MRSA bacteraemia, despite initially satisfying several features of uncomplicated SAB, was associated at autopsy with occult infection involving a recently implanted DES. Uncertainty regarding whether a recently implanted coronary stent should be considered non-removable intravascular prosthetic material may therefore affect SAB risk classification and treatment duration.

Negative findings alone cannot exclude CSI. In this case, follow-up blood cultures became negative, inflammatory markers normalized, TTE showed no vegetation, follow-up CAG was unrevealing, and CT identified no infectious focus. Omission of transoesophageal echocardiography (TEE) for infective endocarditis evaluation was consistent with a VIRSTA score below 3 and a non-suggestive TTE.^6^ However, this assessment does not exclude localized CSI. Intermittent negative blood cultures can occur in SAB and do not definitively exclude a localized endovascular focus.^7^ Persistent chest discomfort despite defervescence and normalized inflammatory markers remained the only clinical clue and should lower the threshold for targeted reassessment after recent PCI.

Previous CSI reports commonly describe overt structural complications such as abscess, pseudoaneurysm, perforation, or stent thrombosis.^1–3^ More recent cases suggest that CSI may evolve dynamically: fluorodeoxyglucose positron emission tomography (FDG-PET)/CT may localize stent-associated inflammation before pseudoaneurysm becomes evident on repeat CT, whereas optical coherence tomography (OCT) and IVUS in established CSI have demonstrated severe disruption of normal arterial wall architecture.^8,9^ In contrast, our patient had persistent chest discomfort despite unrevealing follow-up angiography and conventional CT. Electrocardiographically gated coronary CT, FDG-PET/CT, or intracoronary imaging may therefore provide complementary information when suspicion persists. IVUS or OCT on day 28 might have detected peri-stent abnormalities, although it remains uncertain whether they can identify CSI before overt structural disruption.

The principal therapeutic issue was risk classification rather than selection of a specific antimicrobial agent. Uncomplicated SAB requires exclusion of infective endocarditis, non-removable prosthetic material, persistent bacteraemia, and metastatic infection, with prompt defervescence; treatment is generally recommended for at least 14 days, compared with 4–6 weeks or longer for complicated SAB.^4,5^ This episode was considered uncomplicated because fever resolved within 72 h, follow-up cultures and TTE were negative, and no metastatic focus or prosthetic material other than coronary stents was identified. Had the recently implanted DES been regarded as non-removable intravascular prosthetic material, a complicated SAB framework might have prompted prolonged therapy, repeated cultures, and dedicated repeat imaging.

Endothelial and arterial wall injury, together with incompletely endothelialized struts, may facilitate *S. aureus* adherence and subsequent extension into the arterial wall and surrounding tissue.^10^ Infection involved the recently implanted RCA ostial DES, whereas the remote left anterior descending artery stent showed no disruption; this distribution suggests, but does not prove, greater vulnerability of recently implanted devices.

This report is limited by its single-case design and the absence of coronary tissue culture, molecular testing, or 16S rRNA analysis. Although recurrent MRSA bacteraemia and gram-positive cocci with neutrophilic infiltration at the stented segment strongly support MRSA CSI, direct microbiological confirmation and proof that the recurrent isolate was identical to the initial strain were unavailable. TEE, electrocardiographically gated coronary CT, or FDG-PET/CT might have provided further information, but whether they would have established an earlier diagnosis remains uncertain.

In conclusion, this case raises the possibility that a recently implanted DES may serve as an occult endovascular focus during SAB. CSI may not be excluded by negative follow-up blood cultures, sustained defervescence, or unrevealing echocardiography, CAG, or conventional CT. Persistent or recurrent chest symptoms after recent PCI should prompt targeted reassessment, even when the initial course appears uncomplicated.

## Lead author biography


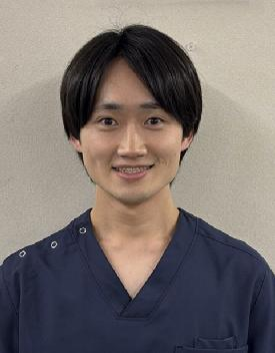


Dr Shota Hirai was born in 1996 and graduated from the Faculty of Medicine, Osaka University, in 2021. After 1 year of specialty training in paediatrics, he decided to pursue adult medicine and entered an internal medicine training programme at Nippon Life Hospital. He is currently a staff cardiologist at Sumitomo Hospital, where he is engaged in interventional cardiology and heart failure care. His clinical interests also include congenital heart disease and cardiovascular genetics.

## Supplementary Material

ytag671_Supplementary_Data

## Data Availability

The data underlying this article are available in the article.
